# High glucose dephosphorylates serine 46 and inhibits p53 apoptotic activity

**DOI:** 10.1186/s13046-014-0079-4

**Published:** 2014-09-27

**Authors:** Alessia Garufi, Gabriella D’Orazi

**Affiliations:** Department of Experimental Oncology, Regina Elena National Cancer Institute, Rome, Italy; Department of Medical, Oral and Biotechnological Sciences, University “G. d’Annunzio”, Via dei Vestini, 31, 66013 Chieti, Italy

**Keywords:** p53, Ser46 phosphorylation, Hyperglicemia, Phosphatase, Calyculin A, Apoptosis, Chemotherapy, Gene transcription

## Abstract

**Background:**

In response to diverse genotoxic stimuli p53 is activated as transcription factor to exert its tumor-suppressor function. P53 activation requires protein stabilization, nuclear localization and posttranslational modifications in key residues that may influence p53 selection of target genes. Among them, serine 46 (Ser46) phosphorylation is considered specific for apoptotic activation. Hyperglicaemia, the high blood glucose condition, may negatively affect tumor response to therapies through several mechanisms, conferring resistance to drug-induced cell death. However, whether high glucose might modify p53Ser46 phosphorylation has never been addressed.

**Methods and results:**

Here, we performed biochemical and molecular analyses in different cancer cell lines treated with chemotherapy in the presence or absence of high glucose condition. Analyses of p53 posttranslational modifications showed that drug-induced p53Ser46 phosphorylation was reduced by high glucose. Such reduction depended by high glucose-induced calyculin A-sensitive phosphatase(s), able to specifically target p53Ser46 phosphorylation. The specific effect on Ser46 phosphorylation was addressed by analysing Ser15 phosphorylation that instead was not modified by high glucose. In agreement, a constitutively phosphorylated Ser46D p53 mutant was resistant to high glucose. As a consequence of phosphoSer46 impairment, high glucose reduced the tumor cell response to drugs, correlating with reduced p53 apoptotic transactivation. The drug-induced apoptotic cell death, reduced by high glucose, was finally restored by the phosphatase inhibitor calyculin A.

**Conclusions:**

These data indicate that high glucose specifically inhibited Ser46 phosphorylation thus reducing p53 apoptotic activity. These results uncover a new mechanism of p53 inactivation providing an interesting novel molecular link between metabolic diseases such as diabetes or obesity and tumor progression and resistance to therapies.

## Background

P53 is the major tumor-suppressor that functions within an extensive signalling network [1]. In response to several types of genotoxic stress p53 is activated to control genes that lead to different cellular outcome such as cell-cycle arrest and apoptosis. In this manner, p53 protects cells from tumorigenesis, reduces tumor progression, and activates tumor cell response to anticancer drugs [2]. P53 activation is achieved at multiple levels including protein stability and sub-cellular localization leading to transcriptional activation of sequence-specific target genes with specific oncosuppressor functions [3]. Apoptosis has been suggested to be a major contribution to p53-mediated suppression of tumor formation [4] and resistance to apoptosis is one of the major hurdles in the treatment of cancer [5]. TP53 posttranslational modifications, such as phosphorylation and acetylation of specific residues, are thought to play a role for the choice among the different biological functions regulated by p53 [6]. It has been proposed that phosphorylation of p53 at N-terminal serine 46 (Ser46) is a necessary step for inducing apoptosis in response to severe DNA damage by shifting from cell-cycle-related to apoptosis-related gene transcription [7,8]. P53 phosphorylated at Ser46 modulates less sensitive gene regulatory elements such as those that control genes encoding proapoptotic proteins including p53AIP1, PIG3, Bax, Noxa, Puma and KILLER/DR5 [7,9-12], or the antiapoptotic factor galectin-3 [13], leading to irreversible apoptosis. To add a layer of complexity, some studies have shown that p53 Ser46 phosphorylation is dispensable for transcriptional activation [14]. However, a defect in Ser46 phosphorylation has been observed in tumor cells that are resistant to p53-mediated apoptosis, and this defect contributes to chemoresistance or the acquisition of the resistance to p53 gene transfer [15]. On the contrary, a mutant active form of p53, in which Ser46 is replaced with phenylalanine (p53-46 F), has been shown to induce apoptosis more effectively that wild-type (wt) p53 [16], while studies with knock-in mice expressing the human *TP53* gene with Ser46A mutation (non phosphorylatable Ser46), reduced p53 apoptotic transactivation [17], strengthening the apoptotic role for this p53 posttranslational modification.

Hyperglicaemia is a pathophysiological condition characterized by high blood glucose concentration that has been shown to predispose to cancer development and progression [18]. Hyperglicaemia is often a consequence of a Western lifestyle that is associated with metabolic syndrome and type-2 diabetes or obesity. Epidemiological evidence suggests that patients with diabetes mellitus are at significantly higher risk of developing many types of cancers [19]. Foods with high glycemic load are most closely correlated with higher recurrence of colon cancer [20]. Moreover, hyperglicaemia may inhibit tumor response to therapies conferring resistance to chemotherapy-induced cell death [21-24]. Glucose metabolism has been shown to reduce p53-dependent transcription of apoptotic Puma gene, although the molecular mechanism of such inactivation was not elucidated [25]. Therefore, in this study we sought to investigate *in vitro* whether high glucose (HG) culture condition might target p53Ser46 in cancer cells and have an impact on p53-induced drug response.

## Materials and methods

### Cell culture and reagents

In this study human lung cancer H1299 (p53 null), colon cancer RKO and HCT116 (carrying wild-type p53), HCT116-p53^-/-^, lung cancer A549 and ovarian cancer 2008 cells (carrying wild-type p53), were used. Cells were routinely cultured in DMEM (Life Technology-Invitrogen) containing 1 g/L D-glucose, supplemented with 10% heat-inactivated fetal bovine serum (FBS) plus glutamine and antibiotics. For high glucose (HG) treatment, cells were transferred to DMEM containing 4.5 g/L D-glucose (Life Technology-Invitrogen), as previously reported [22,23], supplemented with 2% FBS for 24 h before adding chemotherapeutic drugs Adriamycin (ADR) or cisplatin (CDDP) to the culture media respectively at 2 μg/ml and 5 μg/ml for additional 16 h (for ChIP assay) or 24 h (for all the other experiments). Phosphatase inhibitor calyculin A [26] (Sigma) was added at 1 nM along with drugs.

### Viability and tunel assays

For viability assay, subconfluent cells were plated in duplicate in 60 mm Petri dishes and 24 h later transferred to HG medium or DMEM with 1 g/L D-glucose, both containing 2% FBS. The day after, cells were treated with ADR or CDDP for 24 hours. Both floating and adherent cells were collected and cell viability was determined by Trypan blue exclusion by direct counting with a haemocytometer, as reported [27].

Tunel assays were essentially performed as described [28]. Briefly, 4x10^4^ cells were spun on a slide by cytocentrifugation and subsequently fixed in 4% paraformaldheyde for 30 min at room temperature. After rinsing with PBS, the samples were permeabilized in a solution of 0.01% Triton X-100 in sodium citrate for 2 min. Samples, washed with PBS, were then incubated in the TUNEL reaction mix for 1 h at 37°C according to the manufacture’s instructions (Roche, Germany). Cells were counter-stained with Hoechst 33342 before analysis with a fluorescent microscope (Zeiss).

### Chromatin-immunoprecipitation (ChIP) assay

ChIP assay was carried out essentially as previously described [29]. Protein complexes were cross-linked to DNA in living cells by adding formaldehyde directly to the cell culture medium at 1% final concentration. Chromatin extracts containing DNA fragments with an average size of 500 bp were incubated overnight at 4°C with milk shaking using polyclonal anti-p53 antibody (FL393, Santa Cruz Biotechnology). Before use, protein G (Pierce) was blocked with 1 μg/μL sheared herring sperm DNA and 1 μg/μL BSA for 3 h at 4°C and then incubated with chromatin and antibodies for 2 h at 4°C. PCR was performed with HOT-MASTER Taq (Eppendorf) using 2 μL of immuniprecipitated DNA and promoter-specific primers. Immunoprecipitation with non-specific immunoglobulins (IgG; Santa Cruz Biotechnology) was performed as negative controls. The amount of precipitated chromatin measured in each PCR was normalized with the amount of chromatin present in the input of each immunoprecipitation. PCR products were run on a 2% agarose gel and visualized by ethidium bromide staining using UV light.

### Transfection and luciferase assays

Cells were transiently transfected with the cationic polymer LipofectaminePlus method (Invitrogen) according to the manufacturers’ instructions. Luciferase activity was measured in H1299 cells co-transfected with β-galactosidase (β-gal), wtp53 or the constitutively phosphorylated p53Ser46D expression vectors, along with the synthetic PG13-luc reporter or the natural p53AIP1-luc reporter. Luciferase activity was assayed on whole cell extracts and the luciferase values normalized to β-gal activity and protein content and expressed as relative luciferase unit (RLU), as previously described [29].

### RNA extraction and semi-quantitative reverse transcription (RT)-PCR analysis

Cells were harvested in TRIzol Reagent and total RNA was isolated following the manufacturer’s instructions (Invitrogen). The first strand cDNA was synthesized from 2 μg of total RNA with MuLV reverse transcriptase kit (Applied Biosystems). Semi-quantitative Reverse-Transcribed (RT)-PCR was carried out by using Hot-Master Taq polymerase (Eppendorf) with 2 μl cDNA reaction and genes specific oligonucleotides under conditions of linear amplification. PCR products were run on a 2% agarose gel and visualized with ethidium bromide. The housekeeping 28S gene, used as internal standard, was amplified from the same cDNA reaction mixture. Densitometric analysis was applied to quantify mRNA levels compared to control gene expression.

### Western blotting

Western immunoblotting derived from whole cell lysates or nuclear/cytoplasmic extracts was performed as previously described [30]. Total cell extracts were prepared by incubation in lysis buffer (50 mM Tris-HCl, pH 7.5, 150 mM NaCl, 5 mM EDTA, 150 mM KCl, 1 mM dithiothreitol, 1% Nonidet P-40) and a mix of protease inhibitors and resolved by 9-12% SDS-polyacrilamide gel electrophoresis. Proteins were transferred to a polyvinylidene difluoride membrane (PVDF, Millipore) and membranes were blocked with 5% nonfat dry milk in PBS and incubated with the primary antibodies followed by an anti-immunoglobulin–G-horseradish peroxidase antibody (BioRad).

For subcellular fractionation, cells were trypsinized, rinsed with PBS and collected by centrifugation. Cells were then suspended in hypotonic buffer (10 mM HEPES, pH 7.9, 10 mM KCl, 0.1 mM EDTA, 0.1 mM EGTA) and placed on ice for 15 min. NP40 was added to a final concentration of 0.5%. Cells were spun top speed for 30 s before the supernatant (cytoplasmic fraction) was collected. The remaining pellet was washed with hypotonic buffer, resuspended in RIPA buffer, sonicated and spun at 14000 g for 15 min to remove debris and collect the supernatant (nuclear fraction). We confirmed the separation of the cytoplasmic and nuclear fractions by Western immunoblotting of tubulin (cytoplasmic marker) and lamin A (nuclear marker), respectively.

The following primary antibodies were used: mouse monoclonal p53 (DO1) (Santa Cruz Biotechnology), rabbit polyclonal phospho-Ser46 (Cell Signalling and Santa Cruz) and phospho-Ser15 (Cell Signalling), mouse monoclonal anti-poly(ADP-ribose) polymerase (PARP, cleaved form, BD Pharmingen), rabbit polyclonal anti-lamin A and mouse monoclonal anti-β-actin (Santa Cruz), followed by an anti-immunoglobulin-G-horseradish peroxidase secondary antibody (BioRad). Immunoreactivity was detected by enhanced chemiluminescence (ECL) (Amersham GE Healthcare).

### Statistics

Each experiment, unless specified, was performed at least three times. All experiments results were expressed as the arithmetic mean; standard deviation (S.D.) of measurement was shown. Student’s *t* test was used for statistical significance of the difference between groups. Statistical analysis was performed using analysis of variance at 5% (p ≤ 0.05) or 1% (p ≤ 0.01).

## Results

### Glucose reduces chemotherapy-induced cell death

**Figure 1:**
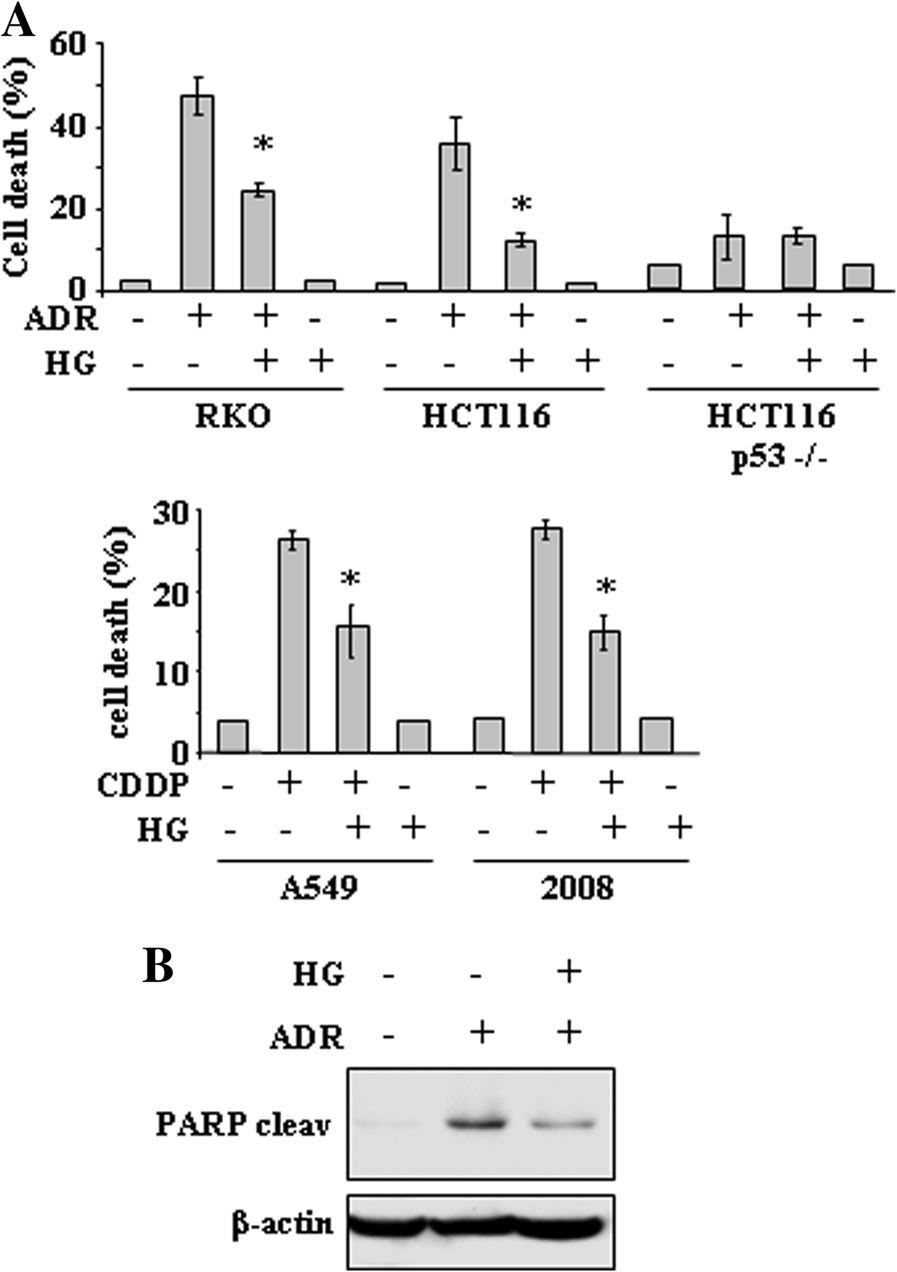
**High glucose (HG) culture condition reduces the cytotoxic effects of drugs in wtp53-carrying tumor cells. (A)** Cells were plated at subconfluence in culture media containing 10% FBS and 1 g/L D-glucose. The day after, medium was changed with a medium containing 2% FBS with either 1 g/L D-glucose or 4.5 g/L D-glucose, respectively low and high glucose (HG), for 24 h before adding chemotherapeutic drugs Adriamycin (ADR) (to RKO, HCT116 and HCT116-p53^-/-^ cells) at 2 μg/ml or cisplatin (CDDP) (to A549 and 2008 cells) at 5 μg/ml. Twenty-four hours later, the percentage of dead cells was scored by trypan blue staining. Error bars show standard deviation. *P = 0.002. **(B)** HCT116 cells were treated with ADR (2 μg/ml) with or without HG culture condition. Equal amount of total cell extracts was analysed by western immunoblotting with anti-PARP antibody specific for the cleaved form (cleav). Anti-β-actin was used as protein loading control.

To look into the influence of high glucose (HG) levels on resistance to chemotherapeutic agents, several human cancer cells, carrying wild-type (wt) p53, including RKO and HCT116 colon cancer, 2008 ovarian cancer and A549 lung cancer cells, were incubated with chemotherapeutic drugs, such as adryamicin (ADR) or cisplatin (CDDP), before or after treatment with HG (4.5 g/L D-glucose) condition. The 4.5 g/L and the 1 g/L D-glucose concentration for, respectively, high glucose (HG) and low glucose condition were chosen according to previous studies [22,23]. Cancer cell death induced by apoptotic doses of chemotherapy, as previously reported [10,31], was significantly reduced when cells were transferred into HG culture condition (Figure 1A). Of note, HG *per se* did not modify cancer cell viability, indicating that it was instead triggering molecular mechanisms of resistance to antitumor drugs. To verify that HG might target p53 activity, HCT116-p53^-/-^ cells were tested in comparison with wtp53-carrying cells. Cell viability assay shows that the slight drug-induced cell death in HCT116-p53^-/-^ cells was not modified by HG condition (Figure 1A), suggesting that p53 inhibition might have a role in HG-induced chemoresistance. Moreover, the ADR-induced cleavage of the apoptotic marker PARP was strongly reduced by HG condition (Figure 1B).

### HG reduces p53 apoptotic transactivation

**Figure 2:**
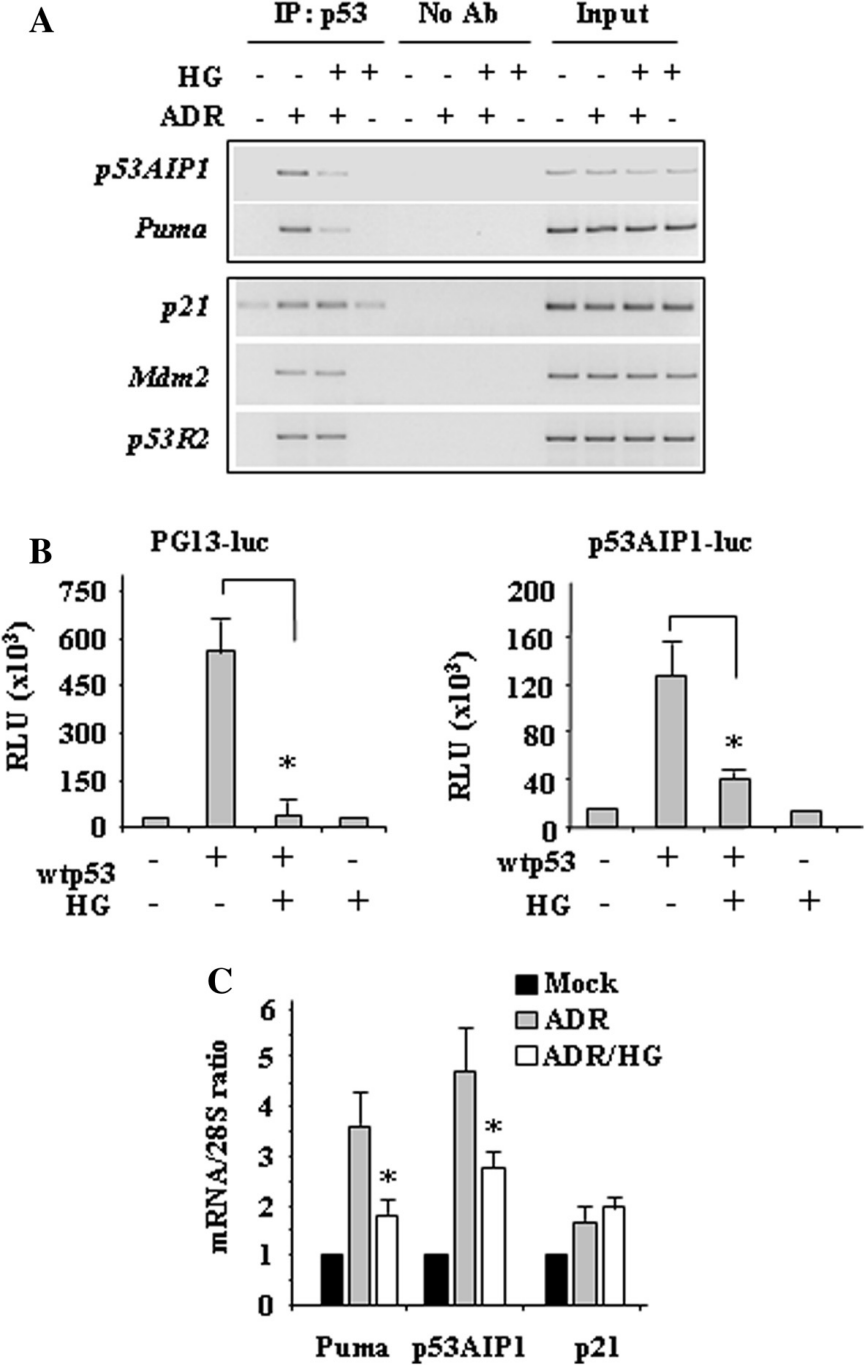
**High glucose (HG) culture condition reduces the p53/DNA binding and transactivation functions mainly on apoptotic genes. (A)** ChIP analyses performed with anti-p53 antibody on HCT116 cells treated with ADR (2 μg/ml) in 2% FBS culture medium with or without HG. PCR analyses were performed on the immunoprecipitated DNA samples using specific primers for the p53 target promoters. A sample representing linear amplification of the total chromatin (Input) was included as control. Additional controls included immunoprecipitation performed with nonspecific immunoglobulins (No Ab). **(B)** H1299 cells were transiently co-transfected with PG13-luc or p53AIP1-luc reporters and wtp53 plasmid. Twenty-four hours after transfection culture medium was changed with a medium containing 2% FBS with or without HG. Results, normalized to β-gal activity are the mean ± S.D. of three independent experiments performed in duplicate. *P = 0.001. **(C)** RNA samples were extracted by HCT116 cells treated as in Figure 1 and used for RT-PCR. The mRNA level of specific p53 target genes was analysed by densitometry and plotted as the expression ratio to control 28S expression. Data are the mean ± S.D. of two independent experiments. *P = 0.001.

Therefore, to evaluate the influence of HG on p53 activity we first analysed the p53/DNA binding activity by ChIP assay and, after that, p53 transactivation. We found that the efficient p53 *in vivo* recruitment onto apoptotic *p53AIP1* and *Puma* promoters in response to ADR was strongly reduced by HG culture condition (Figure 2A). On the other hand, HG did not modify the p53 binding to non apoptotic promoters such as *MDM2*, *p21*, and *p53R2* (Figure 2A). The p53 transcriptional activity was then evaluated by luciferase assay following transient co-transfection of H1299 cells with the synthetic p53 responsive PG13-luc reporter and wtp53 expression vector. As shown in Figure 2B, p53 induced PG13-luc activity that was remarkably reduced by HG condition. In a similar manner, HG reduced the p53-induced transcriptional activity of the natural p53AIP1-luc reporter (Figure 2B). *In vivo* analyses of mRNA levels show that drug-induced upregulation of p53 apoptotic target genes such as *p53AIP1* and *Puma* was strongly impaired by HG condition, while *p21* expression was not modified (Figure 2C). These results indicate that p53 apoptotic transcriptional activity was impaired by HG culture condition.

### HG does not impair p53 nuclear translocation, rather it reduces serine 46 (Ser46) phosphorylation

**Figure 3:**
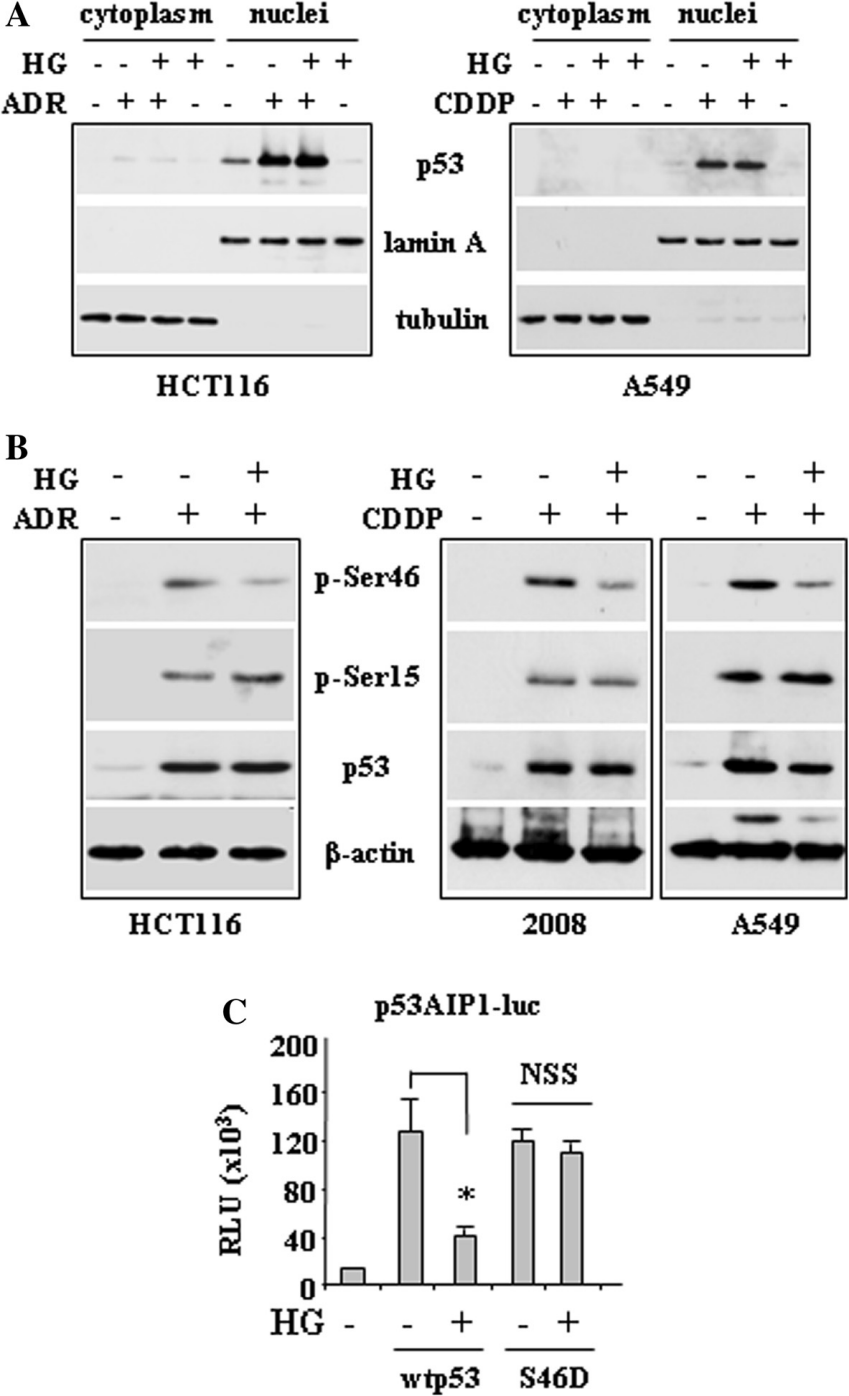
**High glucose (HG) culture condition does not affect drug-induced p53 stability and nuclear localization, rather it inhibits p53Ser46 phosphorylation. (A)** HCT116 and A549 cells were treated with ADR (2 μg/ml) or CDDP (5 μg/ml), respectively, with or without HG culture condition. Equal amount of nuclear and cytoplasmic extracts was analysed by western immunoblotting with specific anti-p53 antibody. Anti-lamin A and anti-tubulin antibodies were used as control of efficient nuclear and cytoplasmic extraction, respectively. **(B)** HCT116, 2008 and A549 cells were treated with ADR or CDDP, as shown, with or without HG. Equal amount of total cell extracts was analysed by western immunboltting with phospho-specific Ser46 and Ser15 antibodies and total p53 antibody. Anti-β-actin was used as protein loading control. **(C)** H1299 cells were transiently co-transfected with p53AIP1-luc reporter and wtp53 or Ser46D mutant plasmids. Twenty-four hours after transfection culture medium was changed with a medium containing 2% FBS with or without HG. Results, normalized to β-gal activity are the mean ± S.D. of three independent experiments performed in duplicate. *P = 0.001; NSS: not statistically significant.

To identify the mechanism underlying impaired p53 apoptotic activity in HG condition, we first examined p53 stability and nuclear localization, which are at the basis of p53 transcriptional activation [6]. Western blotting of nuclear and cytoplasmic cell extracts show that p53 underwent efficient stabilization and nuclear localization following drug treatment, and both conditions were not modified by HG (Figure 3A). These results indicate that the impaired p53 binding to apoptotic promoters following HG, seen above, does not depend on defect of p53 nuclear accumulation. Therefore, we next sought to evaluate whether p53 posttranslational modifications were targeted by HG. In particular, we analysed Ser46 phosphorylation (p-Ser46), since p53AIP1 is a specific target of p-Ser46 [7] and we found above that p53AIP1 expression was inhibited by HG. Western blotting of total cell extracts with phospho-specific antibodies show that drug-induced p-Ser46 was strongly reduced by HG condition (Figure 3B). The specific effect of HG on p-Ser46 was addressed by analysing p-Ser15 that, in comparison, was not modified (Figure 3B). Moreover HG did not alter p53 levels (Figure 3B), indicating that it specifically affected p53Ser46 phosphorylation. In support of our hypothesis that p-Ser46 is a target of HG, we analysed p53AIP1-luc promoter activity following transfection of a constitutively phosphorylated p53Ser46D expression vector. The results show that p53AIP1-luc activity, induced by Ser46D expression, was not inhibited by HG condition, compared to the wtp53-induced p53AIP1-luc activity (Figure 3C). These data indicate that HG specifically inhibited Ser46 phosphorylation though reducing p53-induced apoptotic luciferase activity.

### Inhibition of HG-induced phosphatase(s) by the use of calyculin A restores p-Ser46

**Figure 4:**
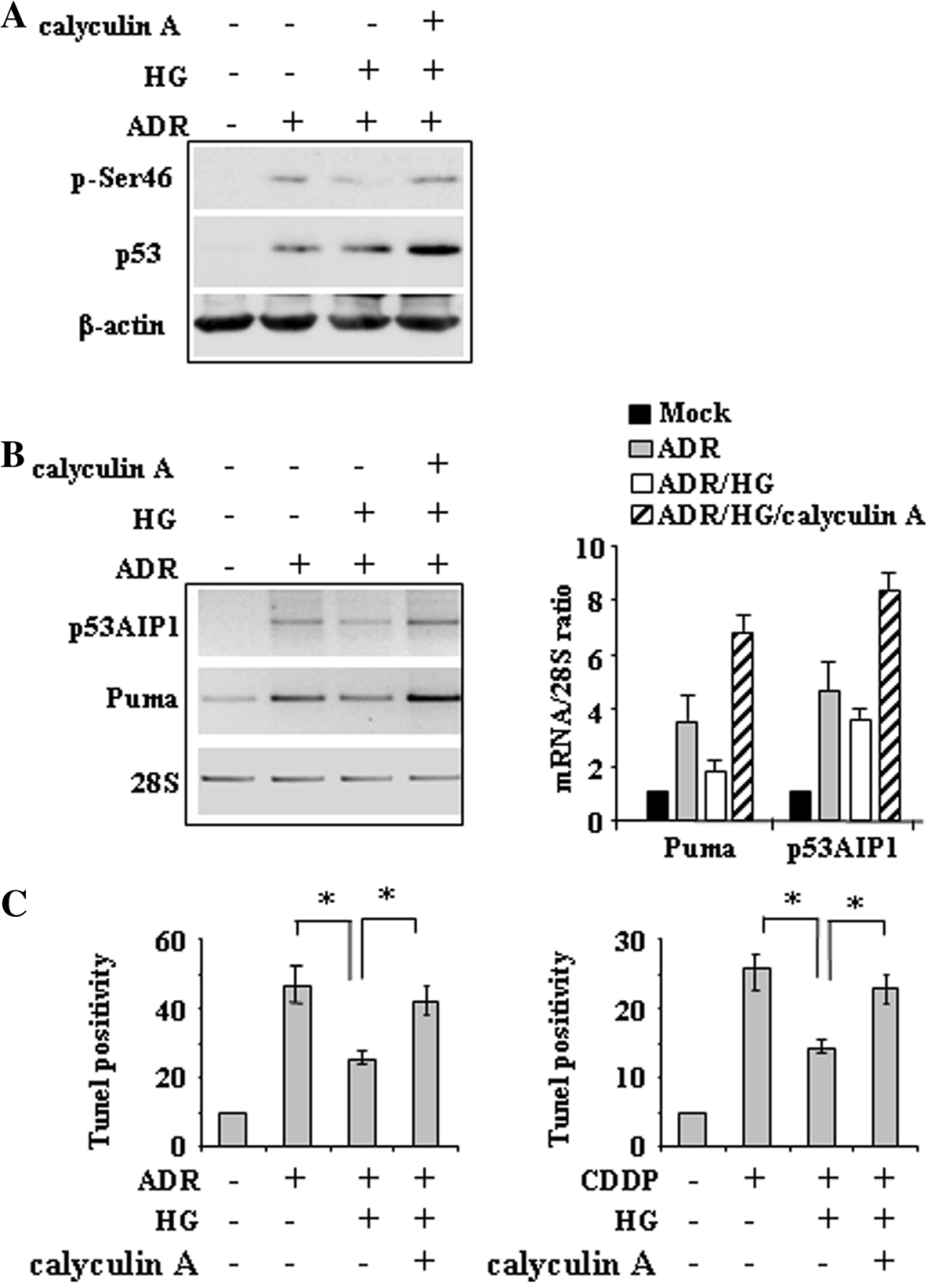
**High glucose (HG) induces calyculin A-sensitive phospahatase(s) able to target p-Ser46. (A)** HCT116, cells were treated with ADR with or without HG; phosphatase inhibitor calyculin A was added at 1 nM along with ADR. Equal amount of total cell extracts was analysed by western immunboltting with phospho-specific Ser46 antibody and total p53 antibody. Anti-β-actin was used as protein loading control. **(B)** RNA samples were extracted by HCT116 cells treated as in (A) and used for RT-PCR of p53 target gene expression. 28S levels were analyzed as internal control (left panel). Densitometric analysis is shown in the right panel and plotted as the expression ratio to control 28S expression. **(C)** Tunel assay of HCT116 and 2008 cells treated with ADR and CDDP, respectively, with or without HG condition. Phosphatase inhibitor calyculin A was added at 1 nM along with drugs. The results shown are the percentage of Tunel positive cells and are representative of two independent experiments performed in duplicates, ± S.D. *P = 0.001.

The above data show that dephosphorylation of p53 at the key Ser46 residue limited p53 apoptotic transcriptional activity. To get insight into the mechanism of Ser46 dephosphorylation by HG we used calyculin A, a cell-permeable phosphatase inhibitor which has been shown to enhance ionizing radiation-induced p-Ser46 [26]. Western blotting show that drug-induced p-Ser46, strongly reduced by HG, was efficiently rescued by calyculin A (Figure 4A). At molecular level, ADR-induced p53 apoptotic-gene expression, inhibited by HG, was rescued by calyculin A (Figure 4B). These data suggest that one or more calyculin A-sensitive phosphatases were triggered by HG to deregulate drug-induced p-Ser46 of p53; however, as calyculin A not only counteracted the negative effect of HG on p53 transactivation but further increased it, it is tempting to speculate the presence of phosphatases also independently of HG condition. Previous studies, including ours, have shown that phosphorylation of p53 at Ser46 activates the apoptotic cascade [7,9,10,14,16], therefore, we investigated the effect of HG on drug-induced apoptotic cell death by Tunel staining. We found that the ADR-induced Tunel positivity was reduced by HG condition and efficiently rescued by calyculin A treatment (Figure 4C). These results indicate that HG dephosphorylated Ser46 in p53 likely through activation of one or more calyculin A-sensitive phosphatases that impaired p53 apoptotic activity.

## Discussion

Here, for the first time a novel glucose-mediated mechanism of p53 inhibition was shown: HG culture condition impaired p53Ser46 phosphorylation and consequently reduced p53 apoptotic activity, attenuating drug-induced cell death. Importantly, the use of the phosphatase inhibitor calyculin A was sufficient to restore Ser46 phosphorylation and drug-induce cell death, indicating that Ser46 dephosphorylation was the mechanism of inhibition of p53 apoptotic activity following HG condition.

In the clinic, the functional status of p53 has been associated with the prognosis, progression, and therapeutic response of tumors [32]. P53 is the most inactivated tumor-suppressor in cancers, therefore, understanding the many ways of p53 regulation is physiologically important because it may affect tumor prognosis and therapy [33]. Thus, many studies in the last years have addressed the reactivation of mutant p53 (see ref. [34,35] and refs. herein) or the reconstitution of wtp53 [36-39] because tumors containing wtp53 are usually more sensitive to radiotherapy or chemotherapy than those bearing inactive p53 [40]. *TP53* is mainly mutated, however, other inactivation involve p53 protein deregulation. In this regard, the present results support a significant conceptual advance in the area of p53 protein inactivation even in the absence of p53 mutations. Human p53 harbors an array of serine/threonine phosphorylation sites that span the entire protein but are mainly concentrated in the N-terminal transactivation domain and the C-terminal regulatory domain Each site/modification may fine-tune p53 function, dictating p53 activity in a promoter-specific manner [41]. Ser46 has recently been the focus of much attention because it has been associated with p53 apoptotic function and its inhibition associated with increased chemoresistance [7-9,15,16]. The functional significance of Ser46 may be also deduced by the codon 47 polymorphism that, although rare, has been shown to interrupt Ser46 phosphorylation and decrease p53-dependent apoptotic activity [42].

Glucose metabolism has been shown to impact p53 function. Glucose restriction can activate p53 apoptotic pathway [43] that is indeed inhibited in cells lacking a functional p-Ser46 [44] and can reduce mutant p53 pro-oncogenic function [45], in line with the beneficial antitumor effect of caloric/dietary restriction [46]. On the other hand, high glucose has been shown to reduce p53-dependent transcription of apoptotic Puma gene, although the molecular mechanism of such inactivation was not elucidated [25]. Here we show that one or more calyculin A-sensitive phosphatases were induced by HG to target p53 at Ser46 thus reducing tumor cell response to anticancer drugs. Further studies will be needed to elucidate the phosphate(s) involved in p53Ser46 inactivation by HG and develop a way to block it, as potential application in clinical practice. In this regard, high glucose has been shown to activate PP2A [47] which has been shown to dephosphorylate p53 at Ser46 [26], making it a good candidate to be analysed in future studies. Moreover, the partial recovery of drug-induced apoptosis following the rescue of p-Ser46, suggests that additional mechanisms of chemoresistance triggered by HG might exist and negatively impact the tumor response to therapies.

In addition, our findings raise more questions for further studies; for example, it would be interesting to investigate whether the HG-induced phosphatase directly dephosphorylates p53Ser46 or rather it targets the upstream kinases claimed to phosphorylate p53 at Ser46, such as homeodomain-interacting protein kinase 2 (HIPK2) [9], ATM [48], DNA-dependent protein kinase [49], protein kinase C δ [50], and dual-specificity tyrosine-phosphorylation-regulated kinase 2 (DYRK2) [51]. Such redundancy in kinases able to phosphorylate p53 at Ser46 support its key role in apoptosis. The possibility that HG might inhibit one of those kinases, rather than targeting directly Ser46, comes from the result that Ser46 phosphorylation is not completely abolished by HG (Figure 3B, Figure 4A). One of the most studied Ser46 kinase, also by us, is HIPK2 which is activated by several cellular stress including chemotherapeutic agents and whose inhibition has been shown to profoundly restrain p53 apoptotic activity. As HIPK2 inhibition impairs Ser46 but also Lys382 acetylation it would be interesting to evaluate the effect of HG on p53 acetylation and its impact on drug response [52,53]. It has been recently shown that reactive oxygen species (ROS) may inhibit HIPK2 kinase activity [54] and, interestingly, a pathophysiological condition that induces ROS production is high glucose [55], strongly supporting more extensive studies about the involvement of HIPK2 in p53Ser46 inactivation following HG condition. In preliminary experiments, thus, we have found that HIPK2 kinase activity was reduced by HG and that also Lys382 acetylation of p53 was impaired by HG (unpublished results).

The acquisition of drug resistance of cancer cells is a big problem in anticancer therapy because it can block apoptotic pathways and/or increase the ability to repair DNA, ensuring the survival of cancer. Understanding the many mechanisms of drug resistance is therefore the subject of intense studies [56]. In this regard, our results show that hyperglycemia may have a negative effect in cancer treatment by inhibiting p53Ser46 apoptotic activity. These results may have clinical impact because highlight how controlling blood glucose levels may have important therapeutic implications in cancer patients.

## Abbreviations

ADR: Adryamicin
CDDP: Cisplatin
ChIP: Chromatin-immunoprecipitation
HG: High glucose
p-Ser46: Serine 46 phosphorylation
RLU: Relative luciferase unit
RT-PCR: Reverse transcription polymerase chain reaction
wtp53: Wild-type p53
